# CYPSI: a structure-based interface for cytochrome P450s and ligands in *Arabidopsis thaliana*

**DOI:** 10.1186/1471-2105-13-332

**Published:** 2012-12-20

**Authors:** Gaihua Zhang, Yijing Zhang, Zhen Su

**Affiliations:** 1 State Key Laboratory of Plant Physiology and Biochemistry, College of Biological Sciences, China Agricultural University, Beijing 100094, People’s Republic of China

**Keywords:** Cytochrome P450, CYP Structure Interface, Template-based modelling, BMCD, ABA 8^′^-hydroxylation, CYP707A

## Abstract

**Background:**

The cytochrome P450 (CYP) superfamily enables terrestrial plants to adapt to harsh environments. CYPs are key enzymes involved in a wide range of metabolic pathways. It is particularly useful to be able to analyse the three-dimensional (3D) structure when investigating the interactions between CYPs and their substrates. However, only two plant CYP structures have been resolved. In addition, no currently available databases contain structural information on plant CYPs and ligands. Fortunately, the 3D structure of CYPs is highly conserved and this has made it possible to obtain structural information from template-based modelling (TBM).

**Description:**

The CYP Structure Interface (CYPSI) is a platform for CYP studies. CYPSI integrated the 3D structures for 266 *A. thaliana* CYPs predicted by three TBM methods: BMCD, which we developed specifically for CYP TBM; and two well-known web-servers, MUSTER and I-TASSER. After careful template selection and optimization, the models built by BMCD were accurate enough for practical application, which we demonstrated using a docking example aimed at searching for the CYPs responsible for ABA 8^′^-hydroxylation. CYPSI also provides extensive resources for *A. thaliana* CYP structure and function studies, including 400 PDB entries for solved CYPs, 48 metabolic pathways associated with *A. thaliana* CYPs, 232 reported CYP ligands and 18 *A. thaliana* CYPs docked with ligands (61 complexes in total). In addition, CYPSI also includes the ability to search for similar sequences and chemicals.

**Conclusions:**

CYPSI provides comprehensive structure and function information for *A. thaliana* CYPs, which should facilitate investigations into the interactions between CYPs and their substrates. CYPSI has a user-friendly interface, which is available at http://bioinfo.cau.edu.cn/CYPSI.

## Background

Cytochrome P450s (CYPs) are heme containing monooxygenases and are found in all eukaryotes. They catalyse various chemical reactions, e.g. hydroxylations, epoxidations, ring extensions and carbon-carbon bond cleavages, and have potential pharmacological and agronomic applications [1-4]. In terrestrial plants, CYPs play important roles in response to biotic and abiotic stimuli by metabolizing a wide range of small organic compounds [5-8]. CYPs are also involved in the biosynthesis of many structural components [9-13].

The three-dimensional (3D) structures of CYPs may provide valuable information that could be used to investigate the interactions between CYPs and ligands. To date, there are more than 5,100 annotated plant CYPs sequences [3,14], but only two have resolved 3D structures (CYP74A and CYP74A2) [15,16]. CYP structures are difficult to determine by standard X-ray or NMR analysis because most of them are membrane-bound proteins. Template-based modeling (TBM) could be a feasible alternative method for obtaining CYP structure information because the 3D structure is highly conserved [1]. There are many choices for CYP TBM, e.g. the class-dependent sequence alignment strategy for CYP TBM [17], SWISS-MODEL [18], MUSTER [19] and I-TASSER [20]. I-TASSER was found to be the most accurate in a recent Critical Assessment of Techniques for Protein Structure Prediction (CASP7-9) [21-23]. However, the models generated by these web-servers have no heme, the position of which is important when investigating the interaction between CYPs and substrates. We developed a pipeline BMCD specifically for CYP TBM (abbreviation of the softwares used: PSI-BLAST, MUSCLE, COMPASS and Discovery Studio 2.1) [24-27].

Most current CYP related resources focus on gene annotation, e.g. the Cytochrome P450 Homepage [28], the CYP engineering database (CYPED) [29] and the Fungal Cytochrome P450 Database (FCPD) [30]. Although some databases collect CYP structure information, e.g. CYPED presents all available 3D CYP structures from the Protein Data Bank [29] and SuperCYP collect many drug-drug interactions and the theoretical models for human CYPs [31], neither of them provide further information about the interactions between ligands and CYPs [29,31].

Our study has developed the CYP Structure Interface (CYPSI), a platform that provides comprehensive structure and function information on all 266 *A. thaliana* CYPs. The models for these CYPs were predicted using the BMCD pipeline and the web-servers: MUSTER and I-TASSER. CYPSI also provides extensive resources for CYPs, including 400 PDB entries for solved CYPs, 48 metabolic pathways associated with *A. thaliana* CYPs, 232 reported CYPs ligands and 18 *A. thaliana* CYPs docked with ligands (61 complexes in total). To demonstrate the quality and utility of the 3D structures in CYPSI, this paper discusses a case study which searches for the candidate CYPs responsible for abscisic acid (ABA) 8^′^-hydroxylation. With the implementation of sequence alignment, the BMCD service for template selection and a structure similarity search facility for small molecules, CYPSI is a comprehensive tool for the investigation of plant CYP structures and functions.

## Construction and Content

### Data collection

The solved CYP structures were collected from the Protein Data Bank (http://www.rcsb.org/) [32]. Up to December 2011, there were 400 PDB entries associated with 76 CYPs (see Additional file 1 for details).

A total of 290 *A. thaliana* CYPs isoforms from 272 CYP genes distributed in 47 CYP families were collected from TAIR10 (http://www.arabidopsis.org/) and http://www.p450.kvl.dk/[33,34], including functional annotations, protein sequences, coding sequences (CDS) and 3,000 base pairs (bp) upstream and 3,000 bp downstream of the CDS.

In addition, 48 metabolic pathways were manually collected from the PMN database [35] and from the scientific literature. Pathways clarified in the scientific literature were marked with “y”, and those that had not been clarified were marked with “na” (see Additional file 2). A total of 232 ligands in these pathways were collected from PubChem [36] or built manually by Discovery Studio 2.1.

### Template-based modelling

BMCD was specifically developed for CYP TBMs, with an emphasis on template selection and sequence alignment. First, profile-profile alignments between the sequence profiles of targets and templates were constructed using COMPASS. Next, the five templates with the smallest evolutional distances (ED) were selected for further TBM. ED was calculated as described in reference [37] using the substitution score matrix, MIYS960102 [38]. Finally, for each target-template pair, three initial models were built using MODELLER in Discovery Studio 2.1 (Accelrys Software Inc.) [27], using the coenzyme heme copied from the template. Of the 15 initial models created for each target, the one with the highest Profiles-3D score was retained for further refinement.

The CHARMm force field in Discovery Studio 2.1 was used by this project for all processes, including energy minimization, molecular dynamic (MD) simulation, the docking program (CDOCKER), and for interaction energy calculations (See Additional file 1 for details).

Besides the BMCD, two servers: MUSTER [19] and I-TASSER [20], were also used for *A. thaliana* CYP model generation by submitting the sequences for *A. thaliana* CYPs manually. The prediction results indicated that out of the 279 *A. thaliana* CYPs longer than 300 amino acids, 266 CYPs would have complete CYP structural domains.

**Figure 1:**
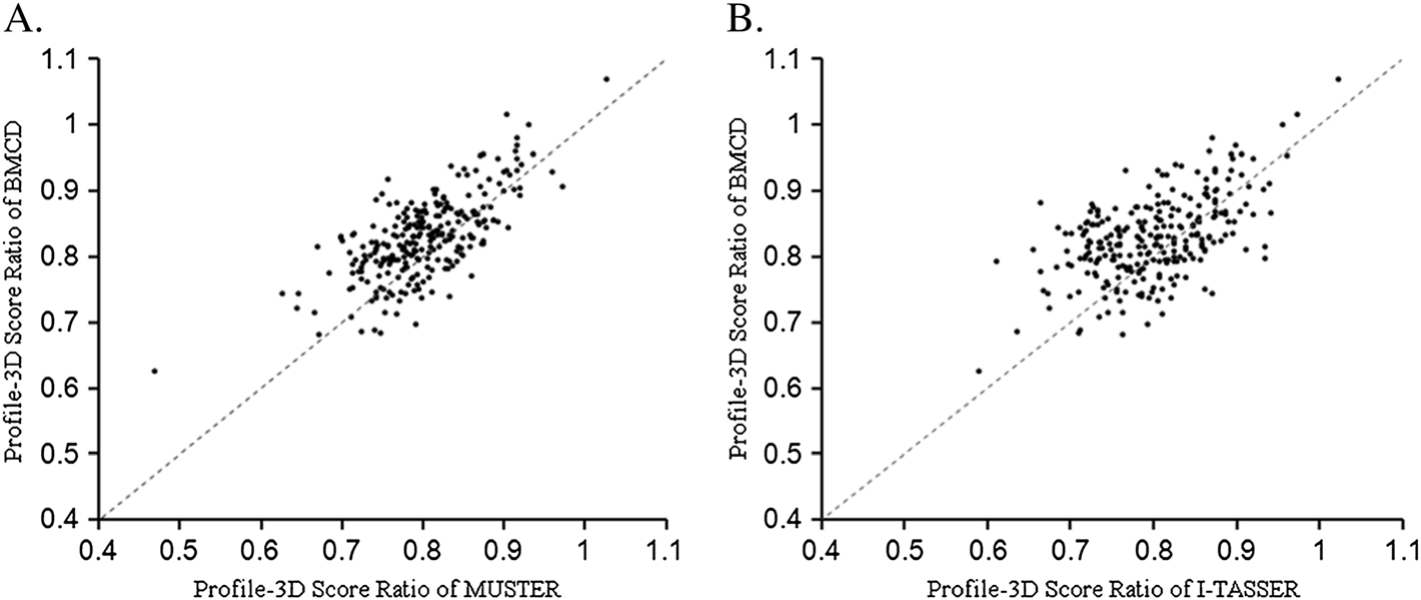
**Models predicted by BMCD have higher Profile-3D Score ratios.** The points above the dotted diagonal line represent models whose Profile-3D Score ratios given by BMCD were higher than the ratios from MUSTER or I-TASSER.

Profile-3D [39] in Discovery Studio 2.1 was used to compare the performance of the three methods and the higher the Profile-3D Score Ratio, the better the 3D structural quality (Figure 1). Paired *t*-test [40] showed that the Profile-3D Score Ratios for the models predicted by BMCD were significantly higher than for the models predicted by MUSTER (P < 2.2e-16) or I-TASSER (P = 7.1e-13). The Profile-3D Score Ratios for the predicted models ranged from 0.75 to 0.95. These ratios were close to those of the solved structures, which ranged from 0.90 to 1.20. This suggested that the model quality for *A. thaliana* CYPs was good enough for practical application.

### A practical application of CYP 3D model

In order to demonstrate the usefulness of the CYP 3D models, a practical application search for CYPs responsible for ABA 8^′^-hydroxylation is presented below.

Firstly, ABA was docked to all nine CYPs candidates (11 models) proposed by Eiji Nambara et al. [5] to be responsible for ABA 8^′^-hydroxylation using CDOCKER [41]. CYP97A3, CYP97B3, CYP97C1 and CYP714A1 were excluded from further analysis because they could not bind ABA and form a suitable conformation for hydroxylation, as determined by our docking result [data not shown].

**Figure 2:**
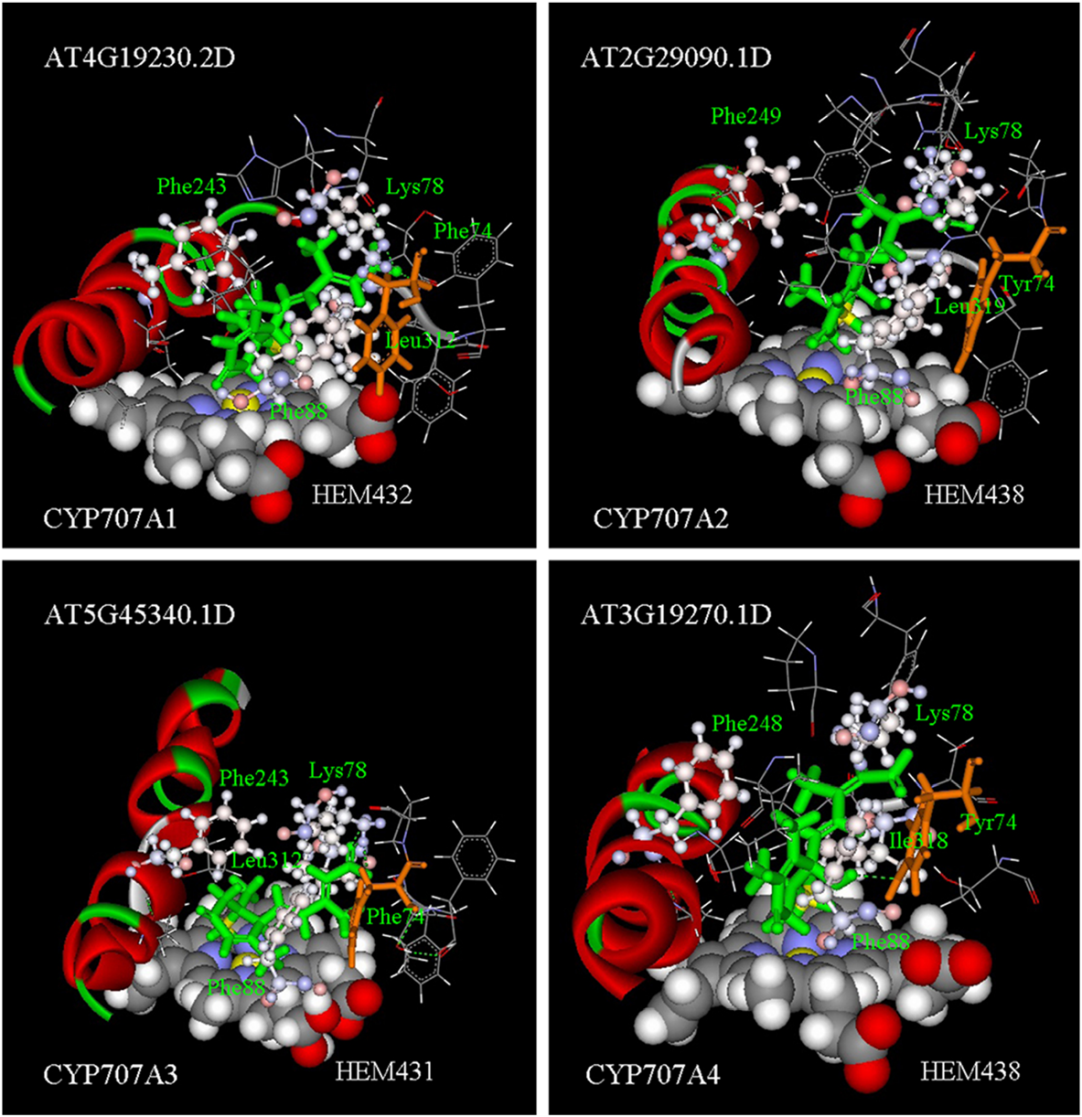
**ABA-CYP707As complexes.** ABA is shown in green and the key residues are shown in “scaled ball and stick” style. Residues: Lys78, Phe88, Phe (around the 245th site) and Ile/Leu (around the 315th site) are important for ABA localization in CYP707As, while residue Tyr74/Phe74 is important for the location of Phe88. The Fe of the heme and the C8 of ABA are shown in yellow. The 74^th^ site is shown in brown.

**Figure 3:**
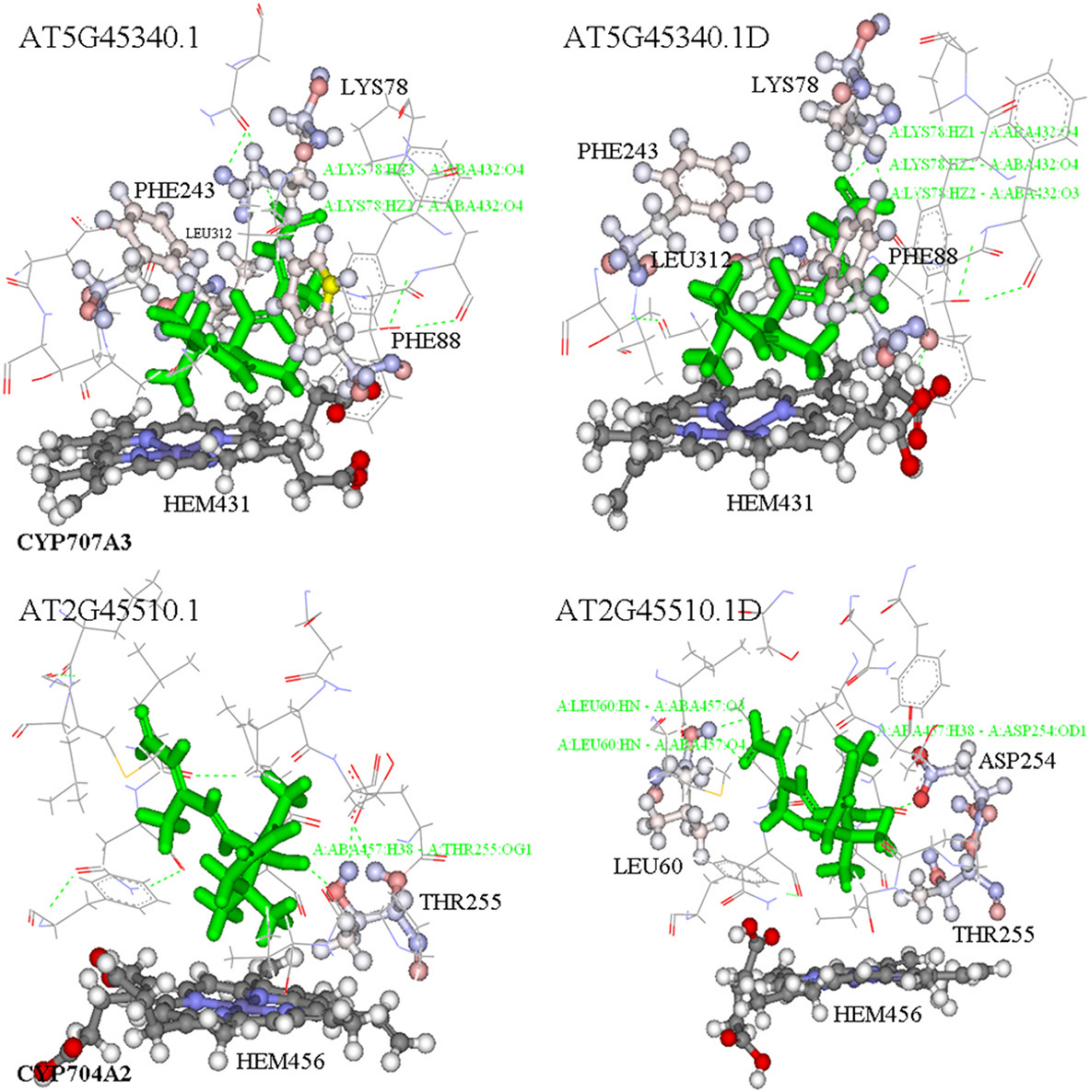
**ABA-CYP707A3 and ABA-CYP7074A2 complexes.** Figures whose AGI names end with “D” represent the last conformation following MD simulation for 50 ps; otherwise the name represents the initial docking complex. The key residues close to ABA are shown in using the ball and stick style. The hydrogen bonds between ABA and residues of the protein are shown by green dotted lines and annotated with bright green words.

Then we examined the key binding residues of the seven initial ABA-CYP complexes for CYP704A2 and six CYP707A proteins (Table 1). The binding sites were similar in all six initial ABA-CYP707A complexes. For example, in ABA-CYP707A3, Lys78 could form a hydrogen bond with ABA; the benzene ring of Phe88 was closely parallel to the ring of ABA; Phe248 had a large contact area with ABA and Leu319 was located between the heme and ABA (Figure 2). However, CYP704A2 lacked the equivalent CYP707A residues needed to firmly bind ABA (Figure 3).

**Table 1:** The Interaction energy (between ABA and receptors) and the Distance (between ABA C8′ and Fe) before and after MD simulation

AGI	CYPs	Interaction Energy (−kcal/mol)			Distance (Å)			Key binding residues
		I	M	MDS ^a^	I	M	MDS ^b^	
AT4G19230.1	CYP707A1	137.279	251.118	272.081	4.174	5.349	4.889	K78, F88, F243 and L312
AT4G19230.2	CYP707A1	216.912	317.753	386.842	3.761	4.401	3.731	K78, F88, F243 and L312
AT2G29090.1	CYP707A2	95.382	315.168	323.671	3.620	4.159	3.804	K78, F88, F249 and L319
AT5G45340.1	CYP707A3	−10.400	281.497	317.584	3.121	3.203	3.574	K78, F88, F243 and L312
AT5G45340.2	CYP707A3	57.678	287.923	320.166	3.445	3.841	4.072	K78, F88, F243 and L312
AT3G19270.1	CYP707A4	166.980	335.875	333.591	4.267	3.399	3.275	Y74, K78, F88, F248 and I318
AT2G45510.1	CYP704A2	−41.931	81.447	108.988	4.315	4.782	6.800	T255

CYP707A1 and CYP707A3 have splicing isoforms. “I”: the initial docking complex; “M”: the complex after three steps of “Minimization”; “MDS ^a^”: the average interaction energy of the conformations following MD simulation; “MDS ^b^”: the last conformation following MD simulation.

Secondly, energy minimization and MD simulation were performed on the seven candidate docking complexes. We compared changes in the ABA locations in these complexes before and after MD simulation. The location of ABA in ABA-CYP704A2 changed considerably compared to ABA-CYP707A, which indicated that this complex was not stable (Table 1, Figure 3 and Additional file 3).

The interaction energy between ABA and CYPs decreased significantly after energy minimization or MD simulation, which indicated that these steps were necessary if a more reliable complex was to be obtained because a lower interaction energy represents firmer binding. It should also be noted that the interaction energy for ABA-CYP704A2 was much higher than that of ABA-CYP707As (Table 1). Integration of the above results, including the binding sites, ABA location and the interaction energy, supported the hypothesis that CYP704A2 is unlikely to be ABA 8^′^-hydroxylase.

CYP707A4 had the lowest catalytic activity for ABA 8^′^-hydroxylation among the four CYP707As [5]. Intriguingly, after MD simulation, a hydrogen bond was formed between the Tyr74 of CYP707A4 and ABA, which did not occur with the other CYP707As (Figure 2 and Additional file 3), possibly because the equivalent residues for the other CYP707As were different from CYP707A4. For example, the 74^th^ residue is Phe for CYP707A1 and CYP707A3. The residue and hydrogen bond differences at the 74^th^ site indicated a lower catalytic activity for CYP707A4 during ABA 8^′^-hydroxylation, which is consistent with previous results [5].

In summary, the docking results suggested that many potential CYPs and key residues should be prioritised for further validation studies (Table 1) and that the results have provided valuable insights into the mechanism behind ABA 8^′^-hydroxylation that need further investigation.

### CYPSI database construction

**Figure 4:**
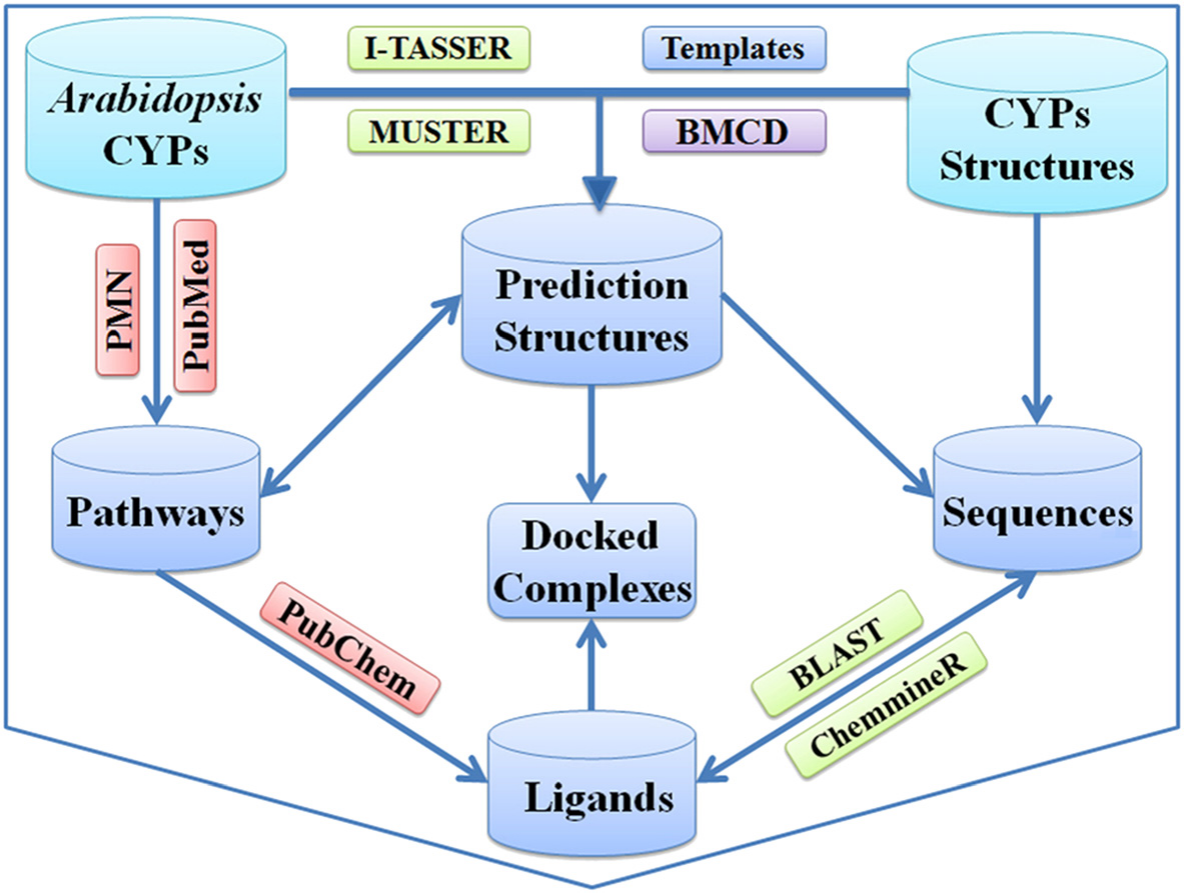
**The CYPSI frame.** The raw data are shown in light blue; the processed data are shown in blue; the utilized tools are shown in green and the data resources are shown in red. The BMCD pipeline for CYP structure modelling was developed as part of this study. “Arabidopsis CYPs”: the *A. thaliana* CYP sequences. “CYPs structures”: the solved CYP structures. “Templates”: the structures recommended as templates for BMCD. “Prediction Structures” were generated by BMCD, I-TASSER and MUSTER. Metabolic “Pathways” were obtained from the PMN database and relevant scientific literature. “Ligands” were collected from “PubChem” or built manually. “Sequences” include the protein sequences of the *A. thaliana* CYPs and solved CYPs. The “Docked Complexes” were generated by CDOCKER software. In addition, BLAST for sequence alignment and ChemmineR for identifying chemicals with similar structures could be used to discover the relationships between CYPs and ligands.

CYPSI was designed as a relational database using a typical LAMP (Linux, Apache, MySQL and Perl) platform aided by JavaScript. An overview of the scheme behind CYPSI is shown in Figure 4 and the relationship among the MySQL tables is shown in Additional file 4. Currently, CYPSI contains six categories of data: solved CYP structures, *A. thaliana* CYP sequences, predicted 3D structures for *A. thaliana* CYPs, related literature, metabolic pathways for *A. thaliana* CYPs and related ligands. In addition, the 18 CYPs that docked with their ligands are also included (see Additional file 5).

Hyperlinks to PDB, TAIR, UniProt [42] and PubMed are provided. Some useful tools are also integrated into CYPSI to facilitate the browsing and search functions, including sequence alignment, a search function for chemicals with a similar structure and 3D structure animation using Jmol [43].

## Utility

### Solved CYPs structures

**Figure 5:**
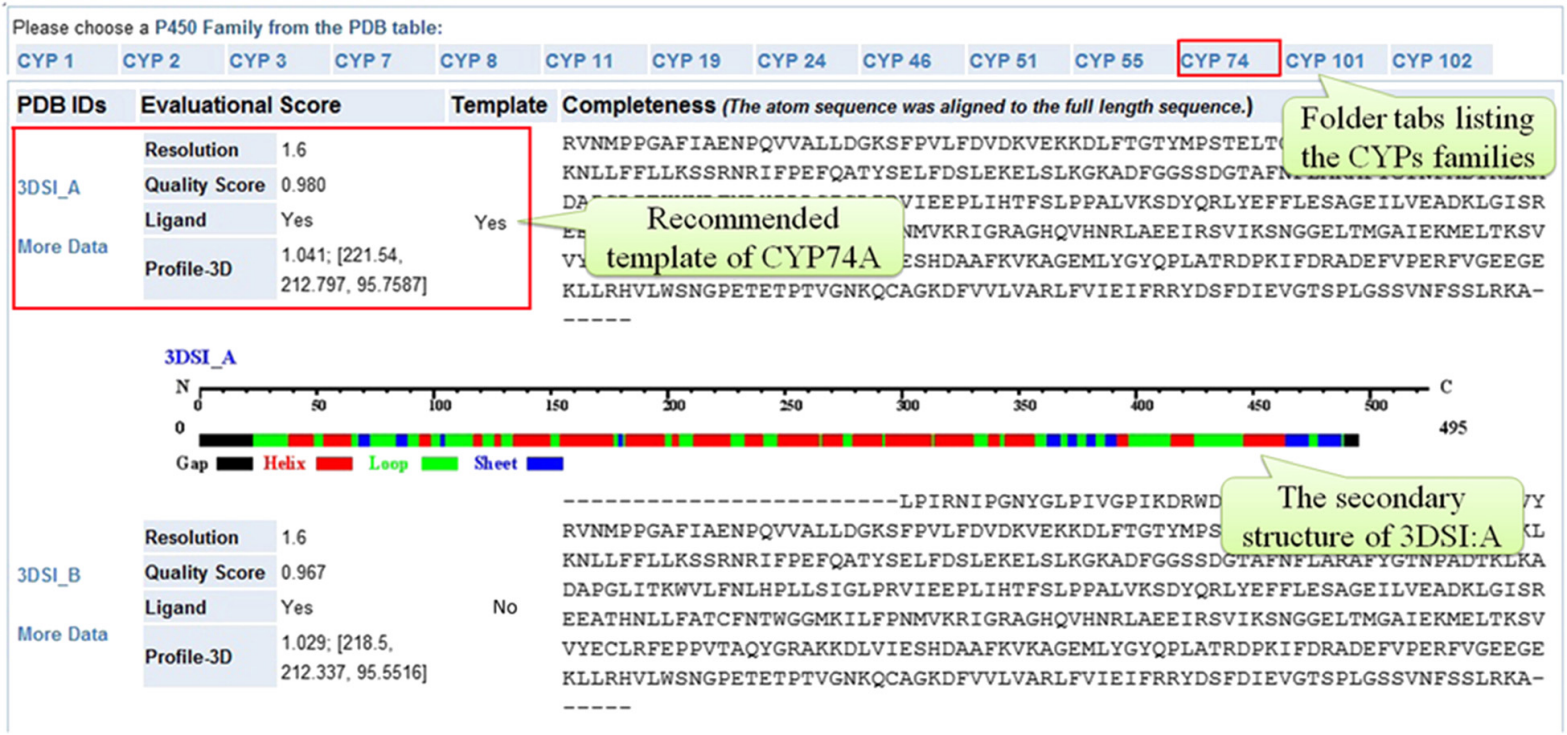
**Structure quality evaluation.** There are 7 PDB entries and 14 structures associated with CYP74A. “3DSI: A” was selected as the template since the “Quality Score” of this complex was the highest, based on structural completeness and the “Profile-3D Score Ratio” (labelled by the red box).

CYPSI contains 689 solved CYP structures associated with 400 PDB entries and provides comprehensive information on protein sequences, secondary structures, ligands and the interactions between ligands and receptors [44,45]. In addition, hyperlinks to PDB, UniProt and PubMed are also provided. For those who wish to perform homology modelling of CYPs, 76 high quality CYP structures, marked with “Recommended” in the “Template” field, are provided (Figure 5).

### *A. thaliana* CYPs models

**Figure 6:**
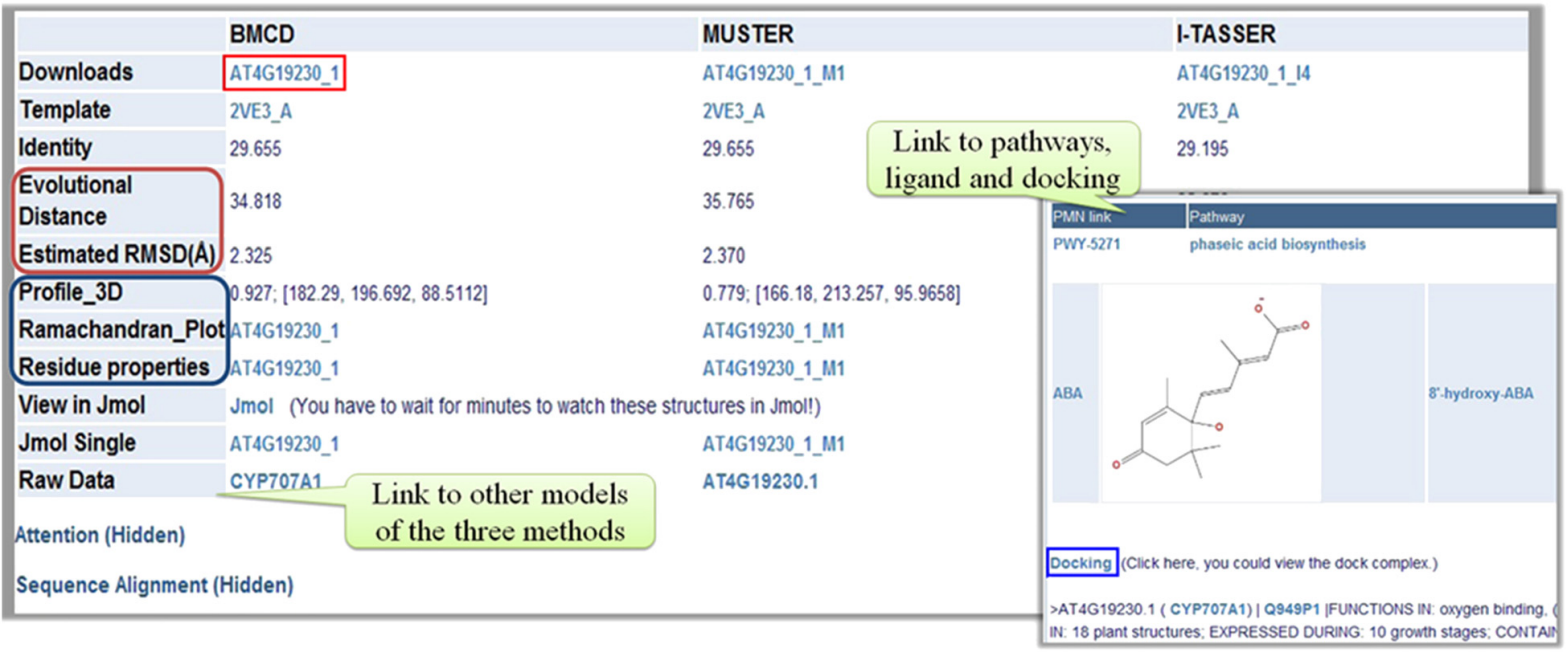
View of the CYP models screen.

The predicted 3D models for 266 *A. thaliana* CYPs are a key feature of CYPSI. Taking CYP707A1 as an example (Figure 6), the best predicted 3D models by the three methods (BMCD, I-TASSER and MUSTER) are shown in a table, which can be used for further research. The model built by BMCD (in the red box) is recommended since it is specifically designed for CYP structure modelling and has been shown to have the best performance. Other initial models predicted by the three methods can be found following the raw data link. The parameters for TBM are provided, including the template, sequence alignment and sequence identity. In order to evaluate the quality of the predicted structure models, the estimated RMSD (in the dark red box), based on the ED of the target and template and the Profile-3D score (in the blue box), are shown. Additionally, links to the metabolic pathways, ligands and docking complexes are supplied if they are in the CYPSI database (located at the lower right corner).

### Metabolic pathways

**Figure 7:**
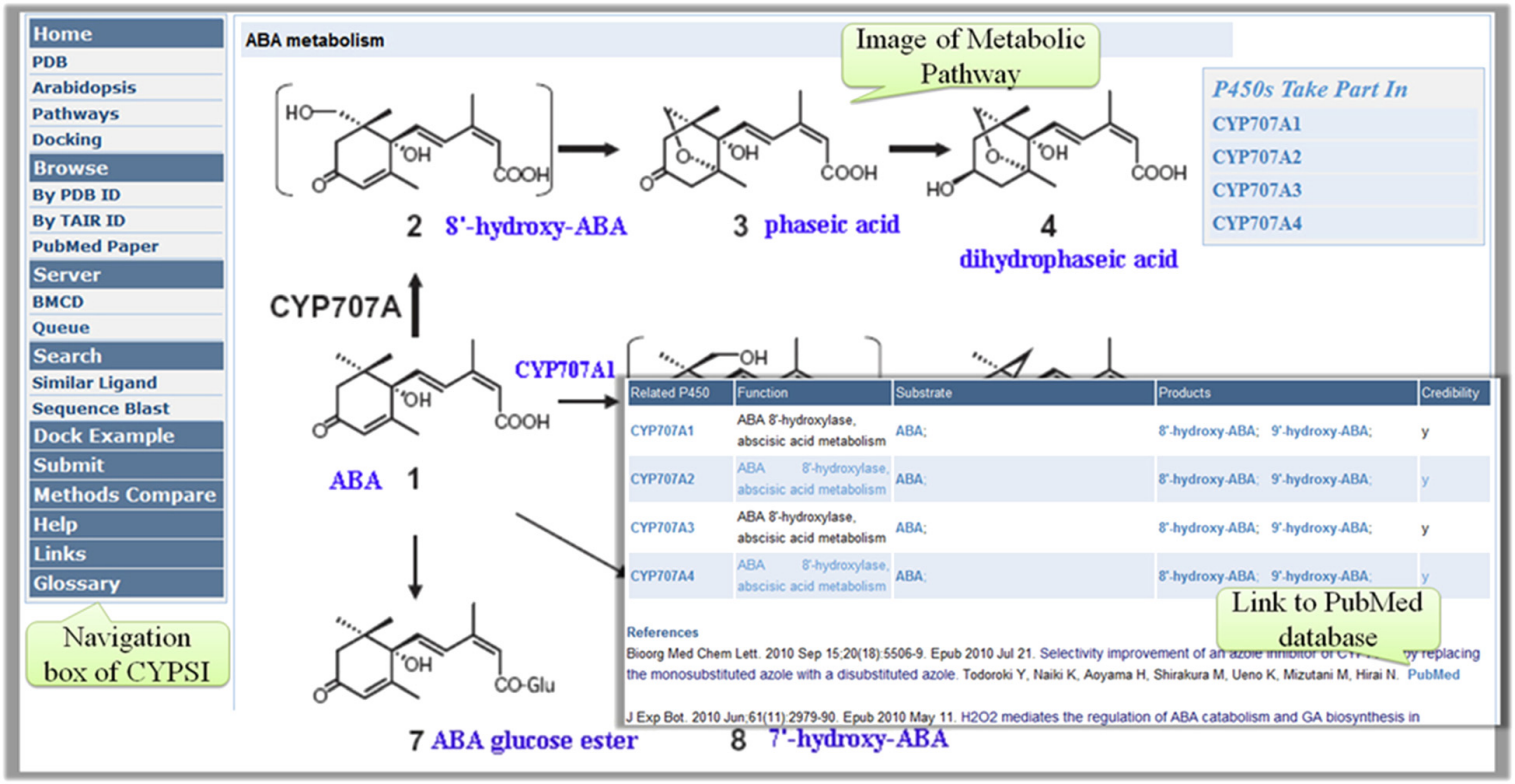
**View of the ABA metabolic pathways screen.** These pathways were collected from the scientific literature with their clarified function marked with a “y” in the “Credibility” field.

Another feature of CYPSI is the comprehensive collection of metabolic pathways and ligands associated with *A. thaliana* CYPs. Around 70 *A. thaliana* CYPs were experimentally investigated, 50 of which have clear functions that are associated with 48 metabolic pathways (see Additional file 2). Figure 7 shows an example page for the ABA catabolic pathway.

### Search capabilities

Besides the ability to browse the data shown above, CYPSI also provides three search capabilities: by keywords, by chemical structures and by protein sequences.

From the search box located at the upper right hand corner of the web-page, users can search for information using the keywords: Arabidopsis Genome Initiative (AGI), PDB IDs, CYP families and pathways.

**Figure 8:**
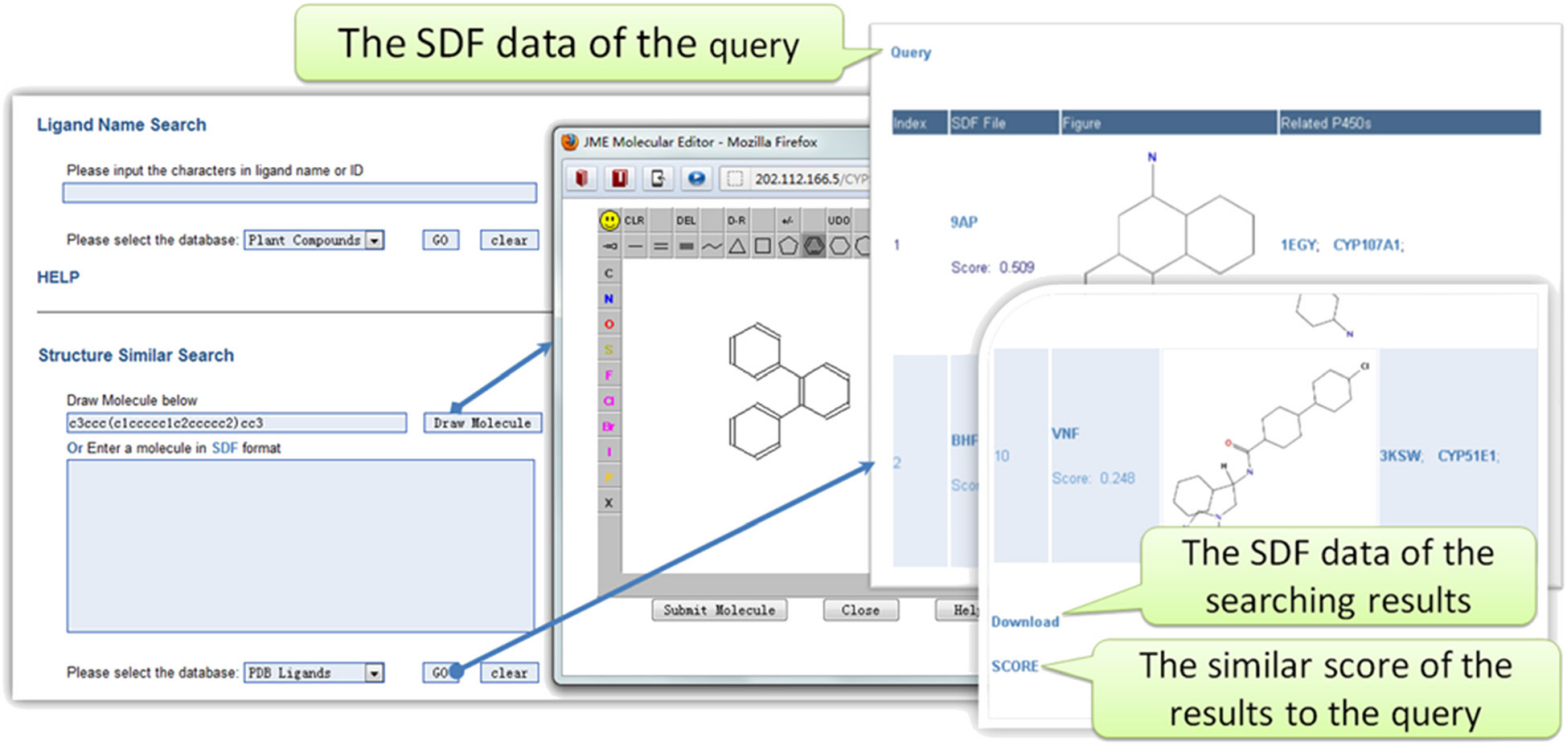
**View of the chemical similarity search screen.** Users can search for a ligand using keywords or structures. Chemical similarity searching is based on ChemmineR. The score is the Tanimoto coefficient.

Figure 8 shows the webpage for chemical structure similarity searches using ChemmineR version 1.4.0 [46]. Users can construct molecular structures online using JME editor (http://www.molinspiration.com/jme/) or submit them in “sdf” format. Version 2.2.20 of the NCBI BLAST algorithm [24] is used for sequence similarity searches (see Additional file 6). In general, CYPs are multi-function enzymes and may have many substrates. In combination with ChemmineR and BLAST, CYPSI could be used to build links between the ligands and sequences of CYPs.

### BMCD server

In CYPSI, the BMCD server is used for template selection and sequence alignment (Additional file 7). Users only need to submit the target CYP sequence and the results will feedback in a few minutes. The sequence alignments given by BMCD can be utilized directly by Discovery Studio 2.1 for TBM.

## Discussion

To facilitate the study of plant CYPs, we have constructed the CYPSI platform, which contains comprehensive information on CYP sequences, structures, ligands and functions. Notably, all *A. thaliana* CYP 3D models were predicted using the BMCD pipeline and preliminary refinements have been made, which is particularly useful when investigating CYP structures and functions. In general, there are four steps involved in TBM: template selection and sequence alignment, model construction, model refinement and model validation.

The quality of the template is a key factor that determines the quality of the predicted models. Prior to TBM, a potential template was carefully selected, taking into consideration the completeness of the structure, resolution, presence of a substrate, and the Profile-3D Score.

CYP sequences are highly diverse and it is hard to find the most suitable template and obtain the correct sequence alignment for TBM [1,17,47]. We developed BMCD for CYP structure modelling and used the profile-profile alignment by COMPASS and ED to evaluate the similarities between templates and targets so that the best template is selected. In addition, most models generated by BMCD are based on a single template as multiple templates may result in considerable structural errors [21,23].

The recommended BMCD models need further refinement, which is even more difficult to control than template selection and sequence alignment [18,21]. Energy minimization and MD simulation are the main methods used for molecular refinement. However, in general, it is difficult to improve the accuracy of the models using these methods [18] as the force fields utilized at present are not accurate enough. For example, in the case of CYP74A modelling (Additional file 8), I-TASSER utilized a special force field to refine the models. However, it performed even worse than MUSTER in terms of RMSD and TM-score [48]. We found that many models, following energy minimization, were worse than the initial BMCD models, as evaluated by the Profile-3D ratio. Therefore, we only refined the residues around the coenzyme heme, which is essential for the study of CYP and ligand interactions.

Despite there being many defects in the field of structure modelling, the CYP models in CYPSI could still be very useful for experimental researchers. In the practical application case study, which searched for CYPs responsible for ABA 8-hydroxylation, although the sequence identities of the CYP707A-template pairs were around 30%, which is theoretically too low to build a high-quality homology model, the docking and MD simulation results coincided well with previous experimental results. These results also identified potential residues for ABA binding, which should help reveal the possible catalytic mechanism involved. However, conformational errors in these models are inevitable. Residues that are close to a ligand may affect the final docking result, so softwares that can cope with both ligand and protein flex are recommended for ligand docking, e.g. AutoDock [49]. Further energy minimization or MD simulation methods are recommended so that more comprehensive and reliable information about the enzyme-ligand complex can be obtained.

## Conclusions

CYPSI was constructed as a comprehensive platform, integrating sequences, structures, ligands and functional information for CYPs. In addition, it also provides useful tools and resources for CYP structural and functional investigations. The recommended models in CYPSI could be used directly for substrate docking and these enzyme-ligand complexes could provide valuable insights for experimental scientists. Further development of CYPSI will lead to the identification of more enzyme-ligand complexes.

## Availability and requirements

The database is available at http://bioinfo.cau.edu.cn/CYPSI, which is compatible with most modern web browsers. All the data in CYPSI are downloadable and freely available to the academic community.

## Abbreviations

CYP: Cytochrome P450; CYPSI: CYPs structure interface; BMCD: PSI-BLAST, MUSCLE, COMPASS, Discovery Studio 2.1; MD simulation: molecular dynamic simulation; ABA: abscisic acid.

## Competing interests

The authors declare no competing interests.

## Authors' contributions

ZS conceived and supervised the study. GHZ developed and tested the performance of BMCD and predicted the structure models. YJZ contributed to the web interface design and the implementation of the tools for search, alignment and structure animation. GHZ, YJZ and ZS collected resources, constructed the database and prepared the manuscript. All authors read and approved the final manuscript.

## Supplementary Material

Additional file 1 **Methods.** Data collection and analysis, including the construction of a sequence profile for the BMCD pipeline, refinement of the initial *A. thaliana* CYPs model, docking, minimization and molecular dynamic (MD) simulation. Click here for file

Additional file 2: Table S1 Metabolic pathways, substrates and products of the *A. thaliana* CYPs. Click here for file

Additional file 3: Figure S1 The complexes formed between ABA and five different CYP707As. Figures whose AGI names end with “D” represent the last conformation following MD simulation for 50 ps. The key residues close to ABA are shown in a ball and stick model. The hydrogen bonds between ABA and residues of the protein are marked with green dotted lines and annotated with bright green words. For CYP707As, the majority of the hydrogen bonds are located between the ABA carboxyl and Lys78. After MD simulation of the ABA-CYP707A4 complex, a hydrogen bond formed between ABA C1’-OH and Tyr74. Click here for file

Additional file 4: Figure S2 A schema for the CYPSI database. Eight MySQL tables found in CYPSI. The arrows represent the relationships between them. Click here for file

Additional file 5: Table S2 The enzyme-ligand complexes and their key residues. Click here for file

Additional file 6: Figure S3 The web interface for sequence similarity searching by BLAST. Click here for file

Additional file 7: Figure S4 The BMCD server. Click here for file

Additional file 8: Table S3 Methods comparison for CYP74A modelling. Click here for file
