# Supplementary material for: CYPSI: a structure-based interface for cytochrome P450s and ligands in Arabidopsis thaliana
Source: BMC Bioinformatics. 2012 Dec 20;13:332. doi: 10.1186/1471-2105-13-332 (PMC3598710; doi:10.1186/1471-2105-13-332)
Supplement: Additional file 1 — Methods. Data collection and analysis, including the construction of a sequence profile for the BMCD pipeline, refinement of the initial A. thaliana CYPs model, docking, minimization and molecular dynamic (MD) simulation. [file 1471-2105-13-332-S1.doc]

###### Additional file 1 - Methods:

## Data collection and analysis

HMMER3 [1] was used to classify the CYPs from the PDB database. CD-HIT [2] was used for clustering the CYPs sequences with a sequence identity of 0.98.

DSSP [3] and LPC [4] were used to analyze the secondary structures and interactions between the ligands and receptors, respectively. Profile-3D software [5] in Discovery Studio 2.1 was used as a statistical criterion to evaluate the structures. The “Profile-3D Score Ratio” equals the “Verify Score” divided by the “Verify Expected High Score” in the Profile-3D software.

To select a template from the solved CYP structures, a “Quality Score” was introduced as it equalled the product of the “Profile-3D Score Ratio” and the “structural completeness”. For the calculation of “structural completeness”, the gap penalty for both ends of the structure was “-1”, and the gap penalty for the interior of the structure was “-10”. They were then divided by the length of the protein.

## Construction of the sequence profile for the BMCD pipeline

The process used for sequence profile construction is described below and it is important for the profile-profile alignment process:

1. In the first step, a curated non-redundant database of CYPs sequences was built. The CYPs sequences were collected from various web sources, e.g. Nelsons Homepage, NCBI, CYPED and Phytozome [6-8]. Then CD-HIT was used to eliminate redundant sequences with a sequence identity of 0.90.
2. The second step was to generate the multiple sequence alignments (MSAs) from a PSI-BLAST search of the query sequence against the above database for six iterations with a threshold e-value of 0.0001.
3. The third step was to build the sequence profile. The evolutional distance (ED) between the query sequence and any sequences in the six MSAs above was calculated based on Equation 1. Using the sorted EDs, from minimum to maximum, and the corresponding sequences in the MSAs above, 150 sequences, whose ED was higher than 10, were collected and ordered. Then they were all aligned to the query sequence using MUSCLE [9]. Finally the sequence profile was built using COMPASS [10].

$ED(S_{x}S_{y})=[1-\frac{2\times\sum_{i}^{n} M_{S_{xi}S_{yi}}}{\sum_{i}^{n} M_{S_{xi}S_{xi}}+\sum_{i}^{n} M_{S_{yi}S_{yi}}}]\times100$ (1)

$M_{S_{xi}S_{yi}}$is the value of the *i*^th^ aligned residues pair in the aligned sequences ***S_x_*** and ***S_y_*** followed by the substitution score matrix, MIYS960102 [11]. The gaps were not considered. $M_{S_{xi}S_{xi}}$and$M_{S_{yi}S_{yi}}$are similar to the$M_{S_{xi}S_{yi}}$, except for the ***i***^th^ site pairs of ***S_x_*** or the ***i***^th^ site pairs of ***S_y_***. ***ED*** *(****S_x_S_y_****)* represents the ED of the aligned sequences, ***S_x_*** and ***S_y_***, respectively. There is an approximately linear relation between ED and RMSD where ED ≈ 21×RMSD-13.50 (the data mining processes are not shown).

## Refinement of the initial *A. thaliana* CYP models

The retained initial models were refined by energy minimization and most of the parameters were defaults [12]:

1. Most of the model atoms were fixed, except for the large loops (A loop length greater than seven amino acids) and for about twelve residues surrounding cysteine that could form a covalent bond with the heme.
2. The bonds between the skeleton atoms of the heme were characterized as “aromatic”.
3. The non-bond cutoff distance was set to 14 Å.
4. The long-range electrostatic interaction was calculated using the spherical cutoff method.
5. The “Smart Minimizer” algorithm was utilized for 2,000 steps.

If no covalent bond (Fe-S) formed between the heme and cysteine, the positions of the residues around cysteine were manually adjusted for further energy minimization or else the initial models would have been regenerated. Some models generated by BMCD used multiple templates.

## Docking, minimization and molecular dynamic (MD) simulation

For ligand docking, after using the program, CDOCKER [13], the active site pocket of the receptor was automatically identified by Discovery Studio 2.1. The site sphere radius was set to 9 Å in order to assign the entire ligand binding pocket. Other parameters were set as defaults. The top 50 docking poses, ranked by the corresponding values for CHARMm energy [12], were preserved in order to identify the most probable binding mode.

Molecular dynamic simulation can provide additional criteria for assessing the robustness of the substrate-enzyme complex and the model. In addition, it could provide more dynamic process information about the interactions between the enzyme and substrate. Energy minimization and MD simulation were performed and were initiated using some of the selected initial complexes constructed above [12]:

1. First, a 7 Å solvation shell with salt ions (0.145mol/L NaCl) around the complex was added.
2. Second, the complex was fixed for energy minimization with the maximum number of steps set to 2,000 in order to allow the best hydrogen-bonding network to form within the solvation.
3. Third, the backbones of the protein were constrained for energy minimization, with the maximum number of steps set to 2,000, in order to allow the best hydrogen-bonding network to form between the side chain and the solvation.
4. Fourth, energy minimization was performed while all atoms were relaxed and the maximum number of steps set at 2,000. The parameters for each step were similar to those used for model optimization by energy minimization.
5. Fifth, unconstrained MD simulation was performed while all atoms were relaxed and most of the parameters used default levels.

Using the complex generated from the energy minimization process, an unconstrained MD simulation was performed and most of the parameters were fixed at default levels.

1. Energy minimization was performed, using a minimum RMS Gradient of 0.10 kcal/(mol·Å), and the maximum number of steps was 5,000 under the Steepest Descent Algorithm.
2. Energy minimization was performed, using a minimum RMS Gradient of 0.0001 kcal/(mol·Å), and the maximum number of steps was 5,000 under the Conjugate Gradient Algorithm.
3. The heating process was performed at temperatures from 50 K to 300 K, using 20,000 steps and a time interval of 0.001 ps.
4. The equilibration process was performed at a temperature of 300 K, using 10,000 steps and a time interval of 0.001 ps.
5. The production process was performed using the Berendsen weak coupling method at a temperature of 300 K. The results were collected every 0.5 ps for 50 ps using a time interval of 0.001 ps.

References

1. Finn RD, Mistry J, Tate J, Coggill P, Heger A, Pollington JE, Gavin OL, Gunasekaran P, Ceric G, Forslund K *et al*: **The Pfam protein families database**. *Nucleic Acids Res* 2010, **38**(**Database issue**):D211-222.

2. Li W, Godzik A: **Cd-hit: a fast program for clustering and comparing large sets of protein or nucleotide sequences**. *Bioinformatics* 2006, **22**(**13**):1658-1659.

3. Hooft RW, Sander C, Scharf M, Vriend G: **The PDBFINDER database: a summary of PDB, DSSP and HSSP information with added value**. *Comput Appl Biosci* 1996, **12**(**6**):525-529.

4. Sobolev V, Sorokine A, Prilusky J, Abola EE, Edelman M: **Automated analysis of interatomic contacts in proteins**. *Bioinformatics* 1999, **15**(**4**):327-332.

5. Eisenberg D, Luthy R, Bowie JU: **VERIFY3D: assessment of protein models with three-dimensional profiles**. *Methods Enzymol* 1997, **277**:396-404.

6. Goodstein DM, Shu S, Howson R, Neupane R, Hayes RD, Fazo J, Mitros T, Dirks W, Hellsten U, Putnam N *et al*: **Phytozome: a comparative platform for green plant genomics**. *Nucleic Acids Res* 2012, **40**(**Database issue**):D1178-1186.

7. Nelson DR: **The cytochrome p450 homepage**. *Hum Genomics* 2009, **4**(**1**):59-65.

8. Sirim D, Wagner F, Lisitsa A, Pleiss J: **The cytochrome P450 engineering database: Integration of biochemical properties**. *BMC Biochem* 2009, **10**:27.

9. Edgar RC: **MUSCLE: multiple sequence alignment with high accuracy and high throughput**. *Nucleic Acids Res* 2004, **32**(**5**):1792-1797.

10. Sadreyev RI, Grishin NV: **Quality of alignment comparison by COMPASS improves with inclusion of diverse confident homologs**. *Bioinformatics* 2004, **20**(**6**):818-828.

11. Miyazawa S, Jernigan RL: **Residue-residue potentials with a favorable contact pair term and an unfavorable high packing density term, for simulation and threading**. *J Mol Biol* 1996, **256**(**3**):623-644.

12. Brooks BR, Bruccoleri RE, Olafson BD, States DJ, Swaminathan S, Karplus M: **A program for macromolecular energy minimization and dynamics calculations**. *J Comput Chem* 1983:4: 187–217.

13. Wu G, Robertson DH, Brooks CL, 3rd, Vieth M: **Detailed analysis of grid-based molecular docking: A case study of CDOCKER-A CHARMm-based MD docking algorithm**. *J Comput Chem* 2003, **24**(**13**):1549-1562.

14. Gaihua. Z, Zhen S: **Inferences from structure comparison: flexibility, secondary structure wobble and sequence alignment optimization**. *BMC Bioinformatics* 2012, **13(Suppl 15)**:S12
