# Supplementary figures and images for: CYPSI: a structure-based interface for cytochrome P450s and ligands in Arabidopsis thaliana
Source: BMC Bioinformatics. 2012 Dec 20;13:332. doi: 10.1186/1471-2105-13-332 (PMC3598710; doi:10.1186/1471-2105-13-332)

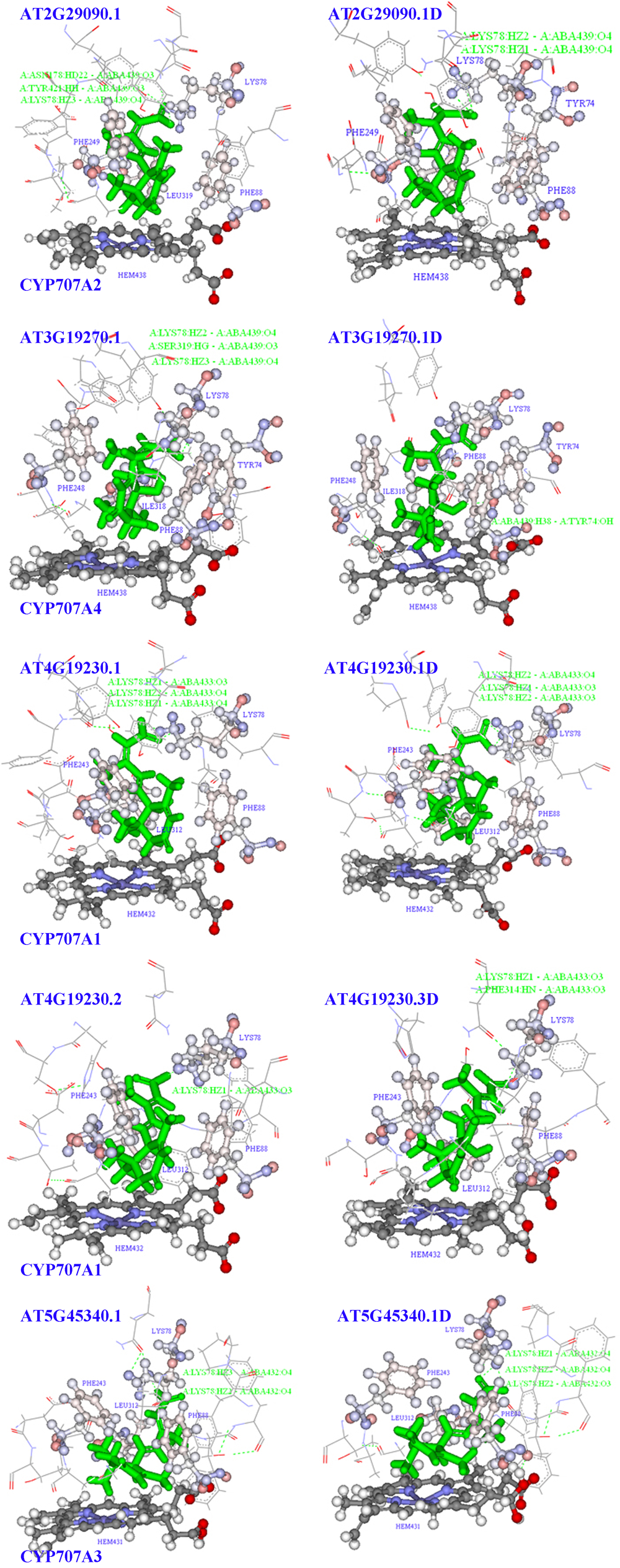

Supplement: Additional file 3: Figure S1 — The complexes formed between ABA and five different CYP707As. Figures whose AGI names end with “D” represent the last conformation following MD simulation for 50 ps. The key residues close to ABA are shown in a ball and stick model. The hydrogen bonds between ABA and residues of the protein are marked with green dotted lines and annotated with bright green words. For CYP707As, the majority of the hydrogen bonds are located between the ABA carboxyl and Lys78. After MD simulation of the ABA-CYP707A4 complex, a hydrogen bond formed between ABA C1’-OH and Tyr74. [file 1471-2105-13-332-S3.tiff]

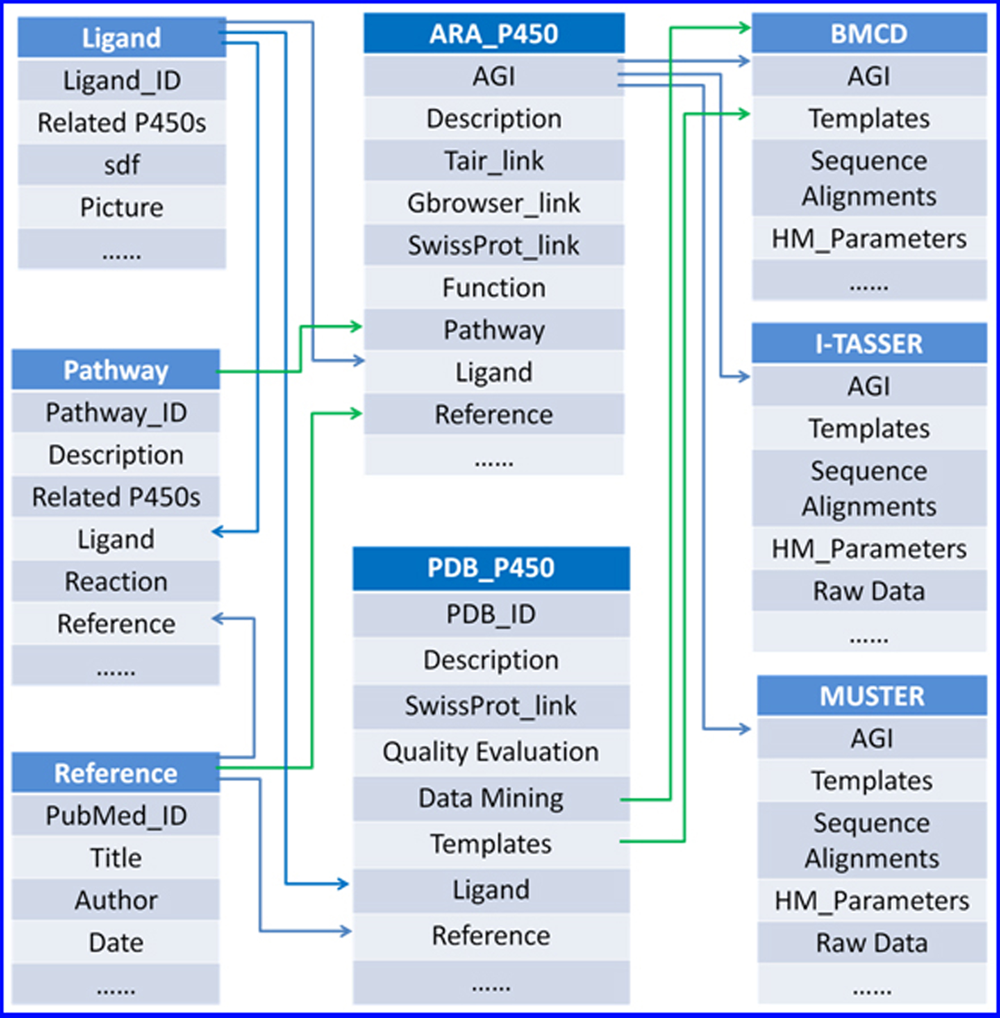

Supplement: Additional file 4: Figure S2 — A schema for the CYPSI database. Eight MySQL tables found in CYPSI. The arrows represent the relationships between them. [file 1471-2105-13-332-S4.tiff]

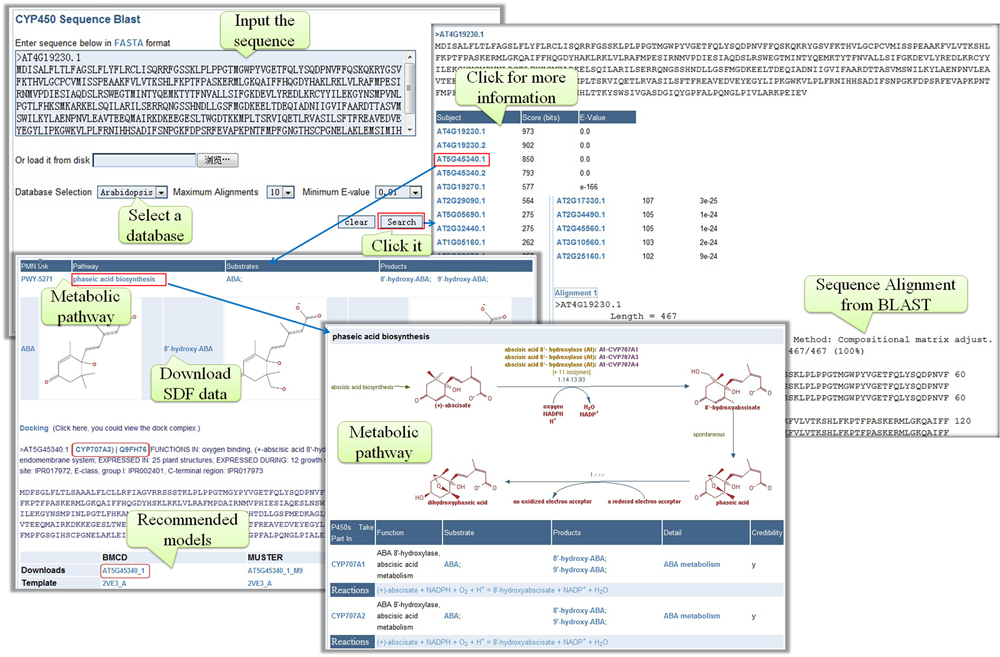

Supplement: Additional file 6: Figure S3 — The web interface for sequence similarity searching by BLAST. [file 1471-2105-13-332-S6.tiff]

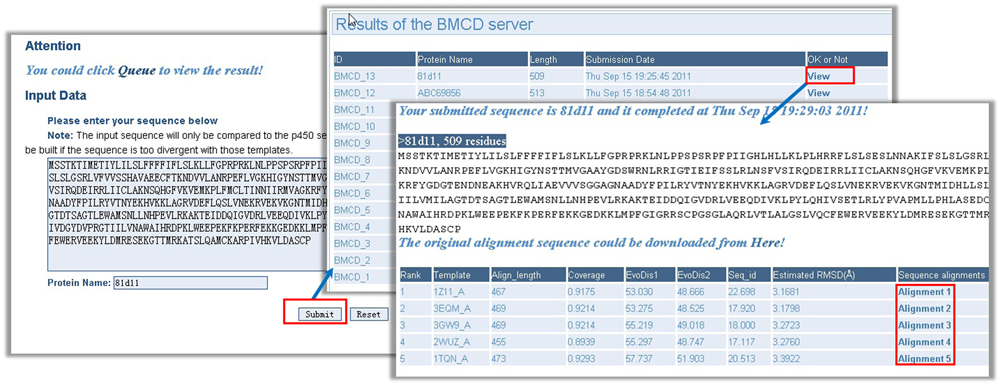

Supplement: Additional file 7: Figure S4 — The BMCD server. [file 1471-2105-13-332-S7.tiff]
