# Supplementary material for: CYPSI: a structure-based interface for cytochrome P450s and ligands in Arabidopsis thaliana
Source: BMC Bioinformatics. 2012 Dec 20;13:332. doi: 10.1186/1471-2105-13-332 (PMC3598710; doi:10.1186/1471-2105-13-332)
Supplement: Additional file 8: Table S3 — Methods comparison for CYP74A modelling. [file 1471-2105-13-332-S8.doc]

###### Additional file 8 - Table S3:  Methods comparison for CYP74A modelling

|  | | **BMCD** | **MUSTER** | **I-TASSER** |
| --- | --- | --- | --- | --- |
| **Template** | | **3DSI:A** | **3DSI:A** | **3DSI:A** |
| Identity (%) | | 100.000 | 100.000 | 99.800 |
| Evolutional Distance (ED) | | 0.000 | 0.000 | 0.000 |
| Profile-3D | Ratio | 1.07 | 0.88 | 0.92 |
|  | Verify Score | 227.50 | 208.68 | 218.13 |
|  | Verify Expected High Score | 212.80 | 236.74 | 236.74 |
|  | Verify Expected Low Score | 95.76 | 106.53 | 106.53 |
| Structure  Comparison | PDB ID | 3DSI: A | 3DSI: A | 3DSJ: A |
|  | Minimum RMSD | 0.12 Å | 0.13 Å | 0.24 Å |
|  | corresponding TM-score | 0.99977 | 0.99972 | 0.99903 |
| **Template** | | **3DAN: A** | **3DAN: A** | **3DAN: A** |
| Identity (%) | | 63.034 | 60.677 | 61.700 |
| Evolutional Distance | | 12.294 | 17.448 | 16.814 |
| Profile-3D | Ratio | 0.98 | 0.80 | 0.89 |
|  | Verify Score | 209.24 | 190.24 | 218.13 |
|  | Verify Expected High Score | 213.72 | 236.74 | 236.74 |
|  | Verify Expected Low Score | 96.17 | 106.53 | 106.53 |
| Structure  Comparison | PDB ID | 2RCL: A | 2RCL: A | 2RCL: A |
|  | Minimum RMSD | 0.87 Å | 0.96 Å | 1.14 Å |
|  | corresponding TM-score | 0.98785 | 0.98111 | 0.97542 |
| In March 2011, CYP74A was submitted to MUSTER and I-TASSER without limitation. In another I-TASSER submission, we limited the template to just “3DAN: A” which was considered to be the best template except itself. The CYP74A sequence was the same as that used in BMCD, which could not model the full length of the models. Then we collected all the models and compared them to all the solved CYP74A structures using TM-align. The minimum RMSD and its equivalent TM-score were collected for the methods comparison [14].  The PDB coordinate data from different experiments may differ considerably because protein structures are flexible. Hence, in this study, all solved structures coordinate data for CYP74A were used for structure comparisons with the predicted models so that the best result could be selected [14]. | | | | |

14. Gaihua. Z, Zhen S: **Inferences from structure comparison: flexibility, secondary structure wobble and sequence alignment optimization**. *BMC Bioinformatics* 2012, **13(Suppl 15)**:S12
